# Highly variable contractile performance correlates with myocyte content in trabeculae from failing human hearts

**DOI:** 10.1038/s41598-018-21199-y

**Published:** 2018-02-13

**Authors:** Michelle L. Munro, Xin Shen, Marie Ward, Peter N. Ruygrok, David J. Crossman, Christian Soeller

**Affiliations:** 1 Department of Physiology, University of Auckland, Auckland, New Zealand; 2 Department of Cardiology, Auckland City Hospital, Auckland, New Zealand; 3 Living Systems Institute & Biomedical Physics, University of Exeter, Exeter, United Kingdom; 4 Present Address: Department of Physiology and HeartOtago, University of Otago, Otago, New Zealand; 5 Present Address: Institute for Experimental Medical Research, University of Oslo, Oslo, Norway

## Abstract

Heart failure (HF) is defined by compromised contractile function and is associated with changes in excitation-contraction (EC) coupling and cardiomyocyte organisation. Tissue level changes often include fibrosis, while changes within cardiomyocytes often affect structures critical to EC coupling, including the ryanodine receptor (RyR), the associated protein junctophilin-2 (JPH2) and the transverse tubular system architecture. Using a novel approach, we aimed to directly correlate the influence of structural alterations with force development in ventricular trabeculae from failing human hearts. Trabeculae were excised from explanted human hearts in end-stage failure and immediately subjected to force measurements. Following functional experiments, each trabecula was fixed, sectioned and immuno-stained for structural investigations. Peak stress was highly variable between trabeculae from both within and between failing hearts and was strongly correlated with the cross-sectional area occupied by myocytes (MCSA), rather than total trabecula cross-sectional area. At the cellular level, myocytes exhibited extensive microtubule densification which was linked via JPH2 to time-to-peak stress. Trabeculae fractional MCSA variability was much higher than that in adjacent free wall samples. Together, these findings identify several structural parameters implicated in functional impairment in human HF and highlight the structural variability of ventricular trabeculae which should be considered when interpreting functional data.

## Introduction

Normal cardiac function is the ability of the ventricles to contract and efficiently pump blood. In heart failure (HF), there is a general loss of cardiac function such that there is an inability to meet the metabolic demands of the body^1^. Causes of human HF are multifactorial and include structural changes that are directly associated with functional deficits. At the tissue level, interstitial fibrosis is widely reported throughout the diseased and failing myocardium^2,3^, leading to an increase in tissue stiffness that opposes active force generation^2^. This fibrosis may occur due to loss of cardiomyocytes (replacement fibrosis), or in the absence of necrotic cell death^3^. In addition to the reduction in proportional cardiomyocyte area as a result of fibrosis, the cardiomyocytes themselves often exhibit sub-cellular structural alterations in HF.

These cellular level alterations often affect components essential for contractile activation and excitation-contraction (EC) coupling, which involves several key structures and proteins within the cell, including the transverse tubules (t-tubules). T-tubules are invaginations of the sarcolemma, creating an intracellular network capable of rapidly conducting action potentials into the cell interior^4^ which is vital for the synchronous activation of Ca^2+^ release from the sarcoplasmic reticulum (SR). Loss or disruption of t-tubules is commonly observed in human and animal models of HF^5–8^ and has been associated with compromised contractile function (development of force) as a result of sub-cellular delays in action potential propagation^9^ and desynchronized Ca^2+^ release^10,11^. Our group has previously identified that the extent of this t-tubule disorganisation is strongly correlated with the severity of cardiac dysfunction in the failing human heart^12^.

The t-tubules normally align to the z-disks^13,14^ which is important for the sarcolemma to closely associate with the terminal SR (jSR) to form junctions^15^. Junctions (also known as dyads in cardiomyocytes) are vital functional regions containing many of the proteins essential to EC coupling, including the SR calcium release channels – the ryanodine receptors (RyR)^16,17^. RyR form clusters at the junction, with their organisation tightly linked to Ca^2+^ handling properties and cardiomyocyte function^18^. Also present is the protein junctophilin-2 (JPH2), which spans the SR membrane and associates with the plasma membrane^19^. JPH2 has subsequently been implicated in the formation and maintenance of both the junctions and t-tubule structure in cardiomyocytes^7,18–21^. In addition, it has previously been suggested that the loss of full-length wild-type JPH2 plays a role in the development and progression of HF^7,22,23^, as well as t-tubule remodeling^20,24–26^. More recently, it has been proposed that microtubule densification (which is observed in HF)^27–29^ is responsible for altered trafficking of JPH2, such that it is displaced from the intracellular junctions, leading to t-tubule disruption and impaired cardiac function^30^.

In order to elucidate the relationship between the various structural changes in HF and the development of contractile impairment, both contractile force and indicators of cell and tissue structure should be examined, ideally within a single muscle sample. Despite the insights provided by correlating ultrastructural remodeling within the cardiomyocytes and the degree of circumferential shortening *in situ* using cine magnetic resonance imaging (cMRI)^12^, cMRI generally lacks cellular resolution and does not allow for the direct measurement of force development. Likewise, isolated cardiomyocytes can be used to examine both cellular function and structure, but they lack information on the role of the extracellular matrix (ECM) and potential fibrosis in HF. An alternative approach is to use cardiac trabeculae which allow examination of cardiac function at the cellular level in the presence of non-myocyte cells (such as fibroblasts) that are present in the ECM and contribute to contractile dysfunction in HF.

Previous studies examining functional alterations in ventricular wall strips or trabeculae from failing human heart found little apparent difference in baseline peak stress development compared to non-failing samples^31,32^. Other studies report peak stress is reduced^33^, or in some instances, increased, in human HF^34^. However, not all studies were performed using physiological temperature or stimulation frequency. Furthermore, many of these studies have been performed in failing patient groups from mixed etiologies, often including ischemic samples, which when sub-divided into cardiomyopathy type reveal different responses^32,35^. This further complicates the interpretation of the underlying mechanisms involved. It is also possible that several studies excluded preparations which demonstrated low or absent stress responses to stimulation. This would lead to experimental selection bias towards ‘healthy’ muscle preparations, despite originating from failing hearts, with some explicitly stating these preparations were excluded from their study^36^. While several previous studies have used cardiac trabeculae to examine functional changes in both human and animal models of HF^2,37–39^, investigation of alterations in trabeculae structure have generally received little attention in those studies. Here, we address this gap and directly examine the relationship between contractile force and structural alterations using ventricular trabeculae from human hearts in failure.

Using a novel approach, we examined cardiac trabeculae from human hearts with idiopathic dilated cardiomyopathy (IDCM) in end-stage HF to directly correlate contractile function (stress measurements) with alterations in cardiomyocyte organisation within the failing heart. Hypothesizing that both tissue and cellular level alterations contribute to the contractile deficit, a range of structural parameters were examined using confocal microscopy. A striking finding was a high degree of variability in the myocyte content of trabeculae which was closely correlated with contractile performance. At the cellular level, trabeculae from failing hearts exhibited microtubule densification which was linked via JPH2 to an increased time-to-peak stress. The highly variable myocyte content of trabeculae excised from the same hearts complicates the interpretation of trabeculae stress measurements, unless these are closely associated with investigation of tissue structure, as shown here, which may explain some of the contradictory results between previous studies.

## Results

### Trabeculae twitch force and variable cardiomyocyte content of human trabeculae

**Figure 1:**
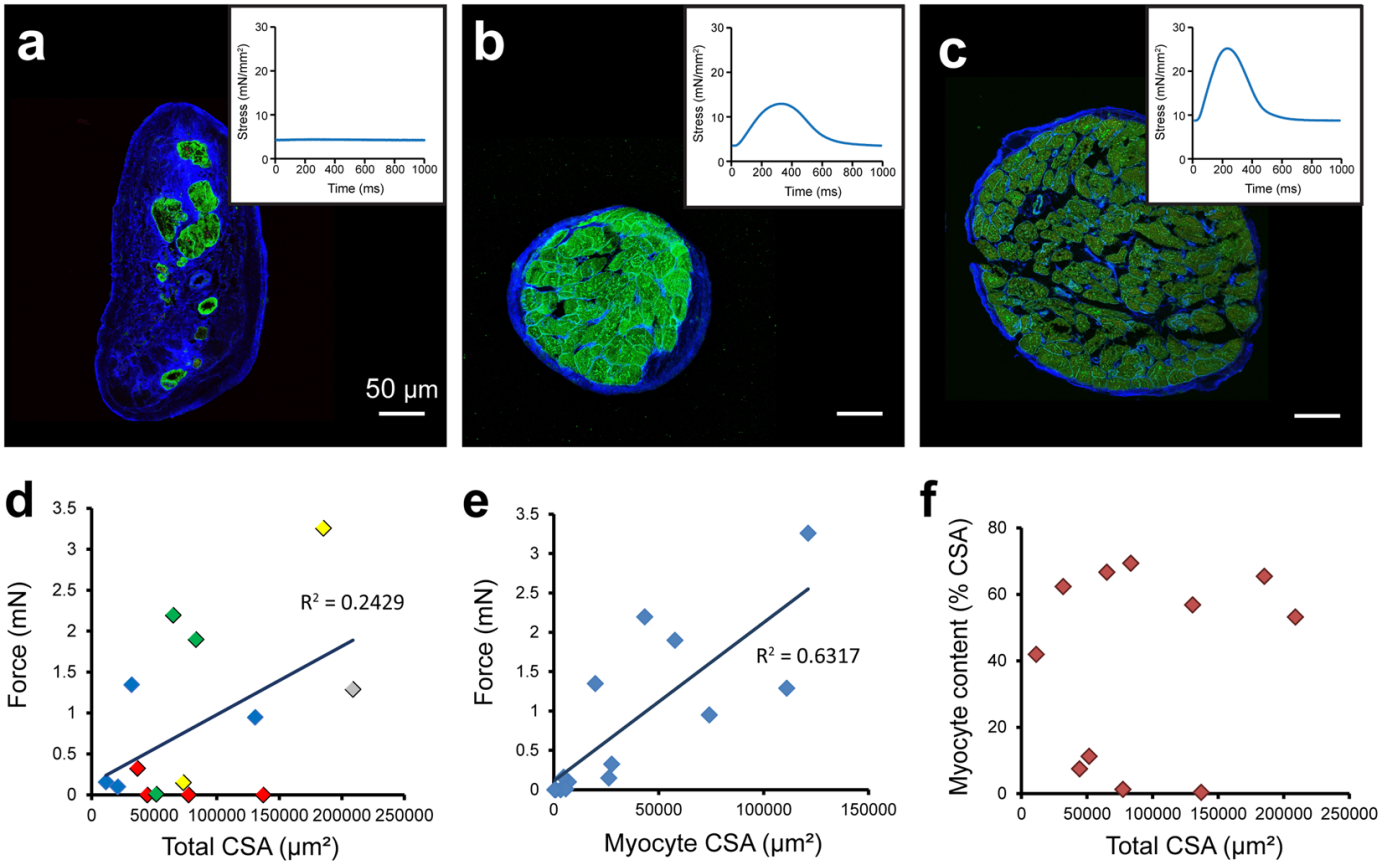
Tissue composition and collagen labelling in failing human trabeculae. (**a**–**c**) Dual labelling of ECM (WGA; blue) and JPH2 (green) in transverse sections of cardiac trabeculae from failing human hearts displaying variable contractile performance. Corresponding stress production at 1 Hz is shown in insets. Active force production forms (**d**) a weak correlation with total trabeculae CSA (p = 0.07), with data points color coded according to patient heart number (DH32: gray, DH36: yellow, DH37: red, DH38: blue, DH40: green), but (**e**) a strong, positive relationship with myocyte CSA in trabeculae from the failing human heart (p = 0.0005). (**f**) No clear relationship was observed between total trabeculae CSA and the percentage of CSA attributed to cardiomyocytes.

Assessment and analysis of force production in the cardiac trabeculae from the failing human hearts revealed a high degree of variability in stress production between samples, when normalised to total CSA (Fig. 1a–c insets show exemplary records). While the mean peak stress developed in these trabeculae (12.8 ± 3.6 mN/mm^2^; see Supplementary Table S1) was similar to that previously reported in IDCM human samples, the stress values developed in some trabeculae from the failing hearts were similar to those reported in healthy human muscle preparations (~23 mN/mm^2^) under similar experimental conditions^33^. It was therefore of interest to determine the key structural parameters contributing to the considerable functional variability observed in these trabeculae. At the tissue level, the composition of the trabeculae was found to be highly variable between samples (Fig. 1a–c), with increasing cardiomyocyte content typically associated with higher peak stress production. Some trabeculae were identified as containing very few, or virtually no cardiomyocytes (Fig. 1a), with large areas of ECM.

The high degree of variability in contractile performance in trabeculae from failing hearts motivated a systematic analysis of trabeculae structure-function relationships. Generally, force generation by trabeculae has been shown to be tightly correlated with CSA of the muscle^40^, however, this was not the case in trabeculae from the endocardial surface of failing human hearts (Fig. 1d). There was also variability in the development of force identified between trabeculae from the same heart (Fig. 1d). This novel observation highlights that while there is inter-heart variability, there is also a high degree of intra-heart variability. Therefore, despite being exposed to the same pre-explantation conditions (such as pharmacological treatments and systemic blood pressure), there is inherent variability in contractile performance of trabeculae isolated from a single failing heart, suggesting that additional, potentially structural, factors contribute to the contractile performance of the trabeculae. It is these factors that we investigate below.

A strong, positive relationship was observed between MCSA and active force production, revealing a statistically significant correlation (Fig. 1e). By contrast, there was no clear relationship identified between the total CSA of the trabeculae and the proportion attributed to cardiomyocytes (Fig. 1f), unlike previous observations in healthy rat hearts^41^. As a consequence, from here on relationships will be presented with stress normalised to MCSA to determine additional structural parameters contributing to the observed variability in force production.

### Variable composition in failing human trabeculae

**Figure 2:**
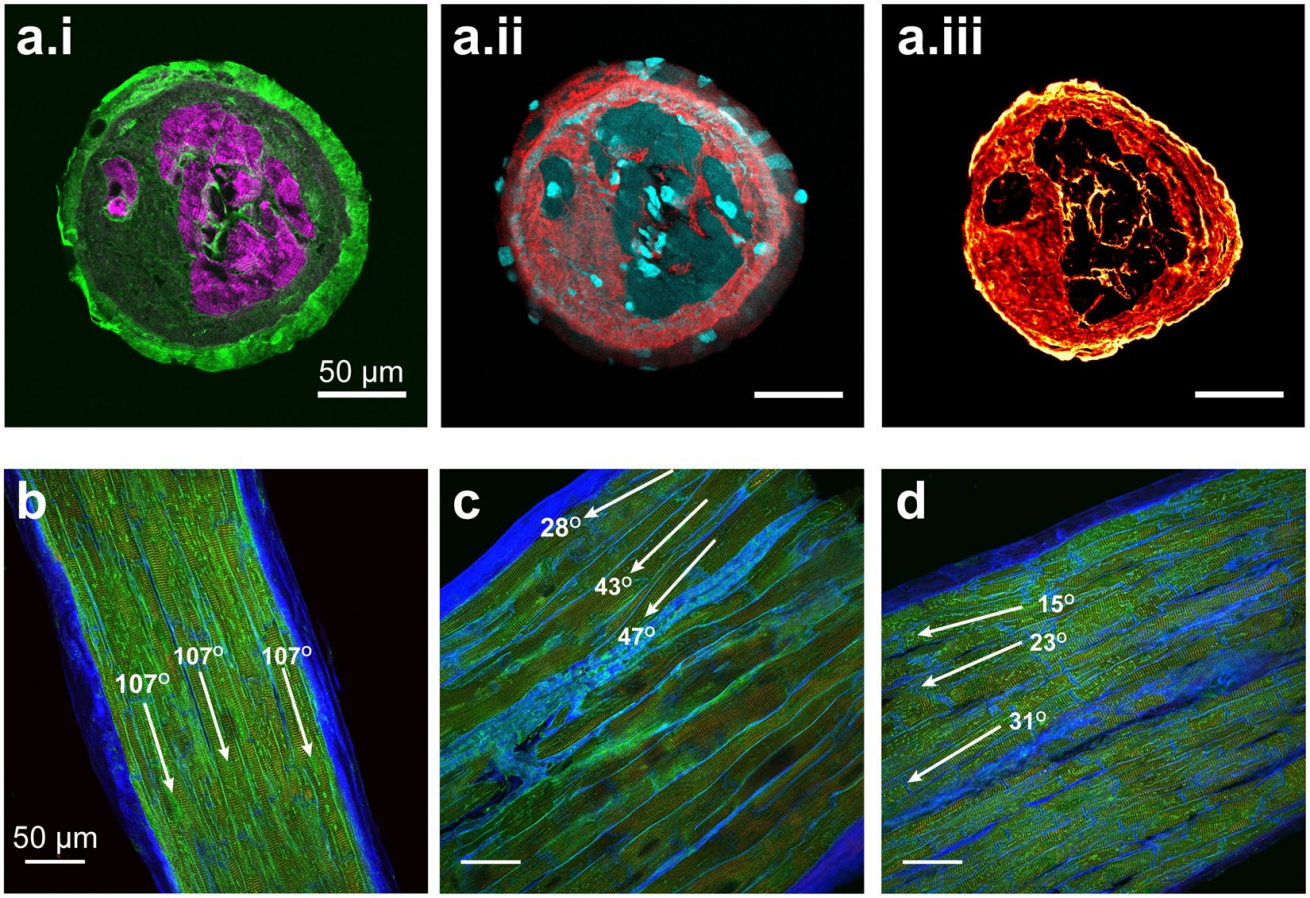
(**a**) Labelling reveals the organisation of (i) phalloidin (magenta) and collagen I (green), (ii) DAPI (cyan) and collagen III (red) and (iii) collagen VI within the same trabecula. (**e**–**g**) Dual labelling of ECM (WGA; blue) and JPH2 (green) in longitudinal sections of cardiac trabeculae from the failing human heart showing different cardiomyocyte alignment.

As identified, there is high variability in cardiomyocyte content in trabeculae from the failing human heart, with some showing extensive ECM. These ECM regions stained positively for collagen I, III and VI (Fig. 2a). Collagen I was particularly strong in the outer-most layer of ECM in the transversely sectioned trabeculae (Fig. 2ai), with both collagen III and VI present throughout the cardiomyocyte-absent spaces (Fig. 2aii-iii). DAPI-positive staining was identified in the extra-cardiomyocyte regions, indicating the presence of additional cell types in the tissue (Fig. 2aii). Cardiomyocytes within trabeculae are considered to be aligned in a parallel manner along the length of the tissue. This was the case for many of the trabeculae in this study (Fig. 2b); however, we also identified that in some trabeculae, there was variability in the angle at which the cardiomyocytes appeared to be running throughout the tissue section (Fig. 2a,d).

### Structural variability in the failing human heart samples

**Figure 3:**
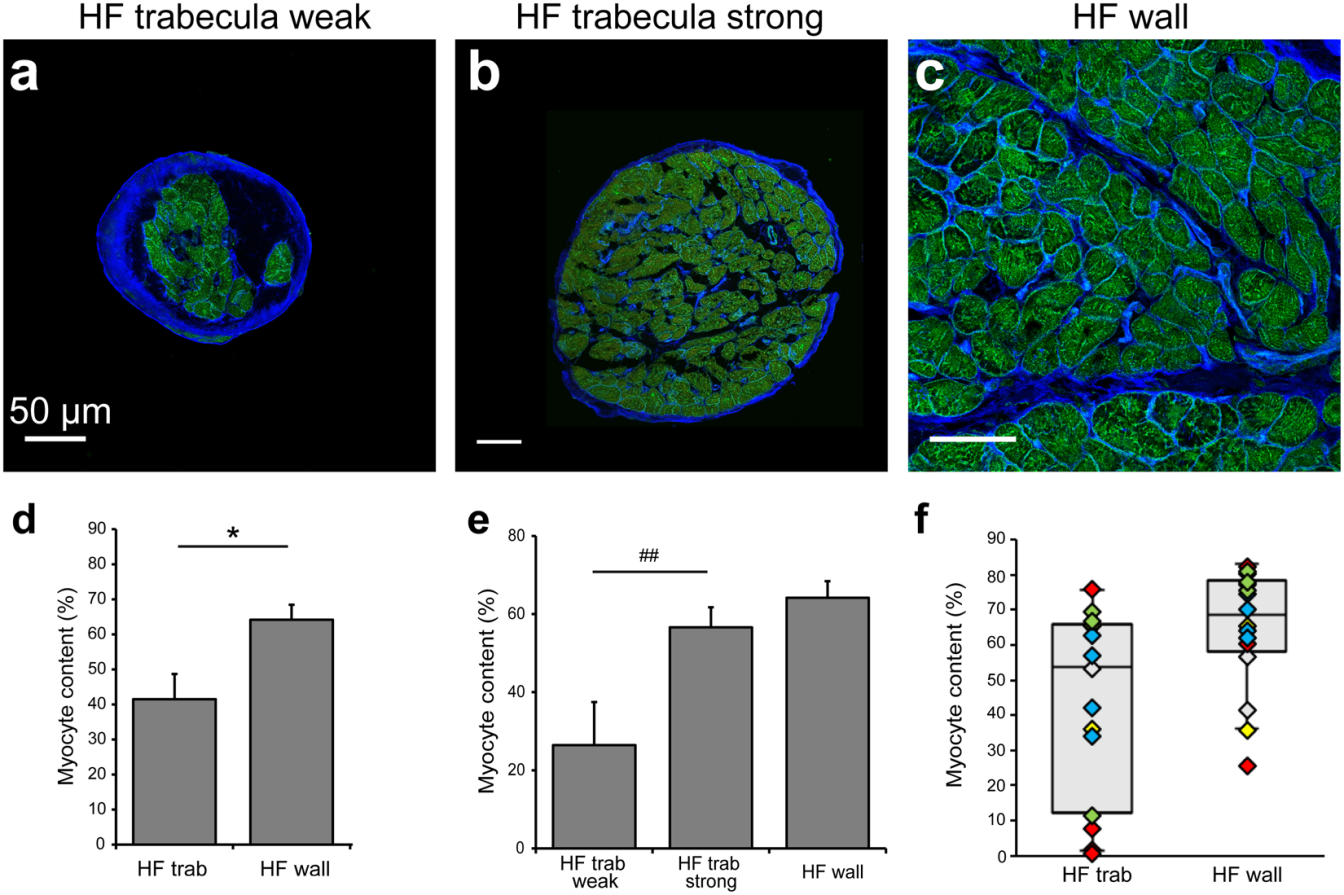
Structural variability and alterations in the failing human heart. Confocal images showing the tissue composition in (**a**,**b**) trabeculae and (**c**) ventricular myocardium from failing hearts with ECM (WGA; blue) and JPH2 (green) labelling. (**d**) Analysis of tissue composition revealed the percentage of tissue attributed to cardiomyocytes in failing trabeculae, failing ventricle wall and (**e**) in failing trabeculae when group according to peak stress development (n = 7 per group). (**f**) Tukey boxplots with data-points color-coded according to individual hearts demonstrates intra-heart variability in trabeculae and wall myocardium from failing hearts (DH32: gray, DH36: yellow, DH37: red, DH38: blue, DH40: green). Myocyte content analysis: Trabeculae: n = 14 images/trabeculae, 5 hearts; failing wall n = 16 images, 5 hearts. Data displayed as mean ± SEM. *p = 0.02; ^##^p = 0.01.

To determine how representative the trabeculae were of the failing myocardium, we also investigated these parameters in samples from the corresponding ventricular wall of the same hearts. In both trabeculae and myocardium from the failing human heart, cardiomyocytes were observed between regions of ECM staining (Fig. 3a–c). On average, the trabeculae from the failing human heart showed significantly reduced myocyte content (41.5 ± 7.2%) compared to their failing ventricular wall counterparts (64.2 ± 4.3%; Fig. 3d). However, when the trabeculae were divided into two groups depending on peak stress development (divided at median; n = 7 per group), the group with higher peak stress showed no difference in mean cardiomyocyte content when compared to the failing ventricle wall, while trabeculae from the group with the lower stress development showed a significant reduction in cardiomyocyte content (Fig. 3e). Further investigation revealed that there was greater intra-heart variability in myocyte content in trabeculae compared to the corresponding ventricular myocardium (Fig. 3f).

### Sub-cellular organisation in human HF

**Figure 4:**
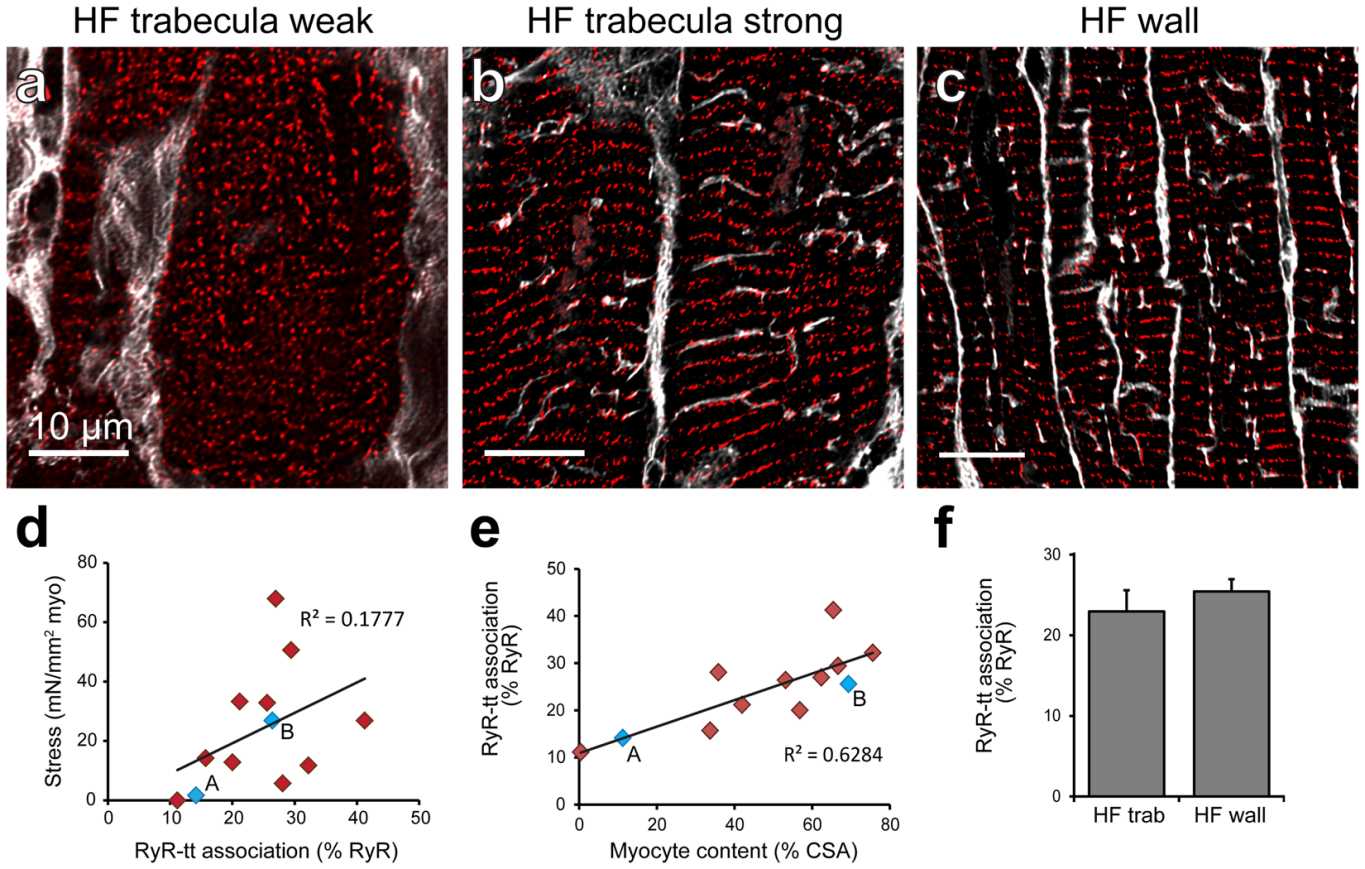
Structural correlates with contractile function in human cardiac trabeculae. Deconvolved confocal images show t-tubule (WGA; grey) and RyR (red) dual labelling in cardiomyocytes from trabeculae exhibiting either (**a**) weak or (**b**) strong contractile function, as well as (**c**) ventricular myocardium from the human failing heart. (**d**) Stress normalised to MCSA forms a weak, positive relationship with the percentage of RyR clusters associated with t-tubule labelling (p = 0.167). (**e**) A strong positive correlation is observed between increasing myocyte content as a percentage of total CSA and the degree of association between RyR clusters and t-tubules (p = 0.0014). Blue data-points labelled ‘A’ and ‘B’ indicate values from corresponding images. (**f**) Analysis of RyR-t-tubule association was performed in the sample groups. RyR-t-tubule association analysis: Trabeculae: n = 22 images, 14 trabeculae, 5 hearts; failing wall n = 28 images, 5 hearts; Data displayed as mean ± SEM.

To identify whether further cellular structural alterations were implicated in the functional variability in the failing human trabeculae, we also investigated sub-cellular organisation of key EC coupling structures – the junctional couplings formed between the t-tubules and jSR (where the RyR are localised). Confocal imaging revealed a high degree of variability in the organisation of both RyR clusters and t-tubules in cardiomyocytes from human trabeculae, with t-tubules virtually absent in some cardiomyocytes (Fig. 4a). The remaining t-tubules often appeared disrupted or occurring at oblique angles (Fig. 4b). Visual inspection of confocal dual labelling of RyR clusters and t-tubules suggested that there was increasing association of these structures in trabeculae exhibiting increased contractile performance (stress normalised to MCSA) and while a weak positive correlation was observed (R^2^ = 0.18), this was not statistically significant (Fig. 4d). Interestingly, a clear positive relationship was identified between increasing cardiomyocyte content of the trabeculae and the proportion of RyR clusters associated with t-tubule labelling (Fig. 4e), suggesting that there is increased preservation of cellular organisation in cardiomyocytes when there is a greater prevalence of myocytes within the tissue.

Regular transverse rows of RyR clusters were observed in ventricular myocytes of failing ventricle wall tissue samples (Fig. 4c), with predominantly longitudinal and oblique t-tubule extensions apparent (compared to transverse organisation in non-failing tissue, see Supplementary Fig. S2) – a feature of structural remodeling which has been regularly reported in HF^5,7,8,24^. These disorganised t-tubules appeared to contribute to the decreased proportion of RyR clusters identified as being associated with t-tubules in the failing wall samples compared to non-failing (Supplementary Fig. S2). There was no difference in the mean percentage of RyR clusters associating with the t-tubule labelling between the trabeculae and ventricle wall samples from failing human hearts (Fig. 4f).

In conclusion, the free wall samples from failing hearts exhibit broadly similar structural changes as have been previously identified as hallmarks of failure. Trabeculae appear similarly affected but present more variable myocyte content.

### Junctional protein co-localisation in the failing heart

**Figure 5:**
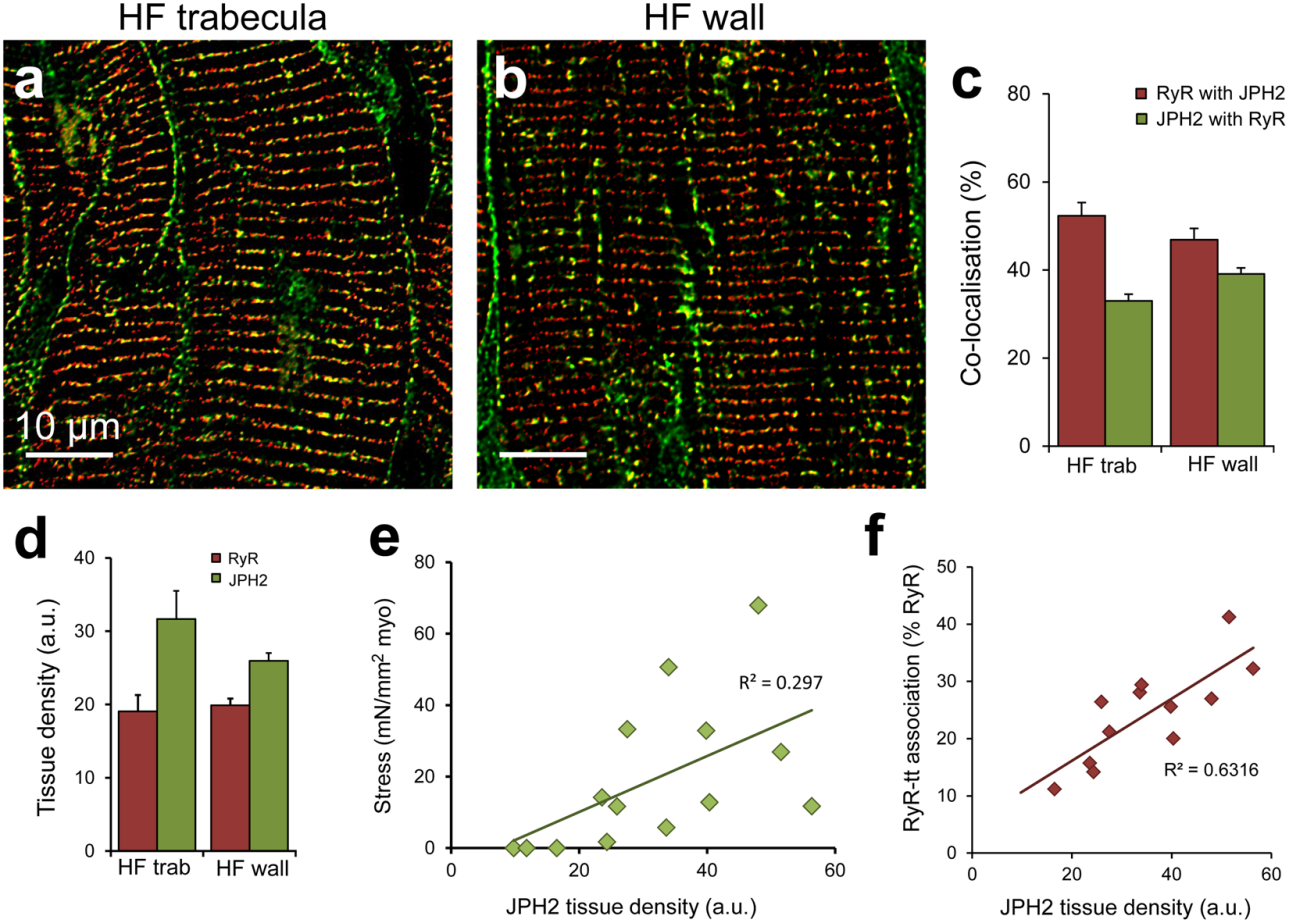
RyR co-localisation with JPH2 is altered in human HF. Deconvolved confocal micrographs showing RyR (red) and JPH2 (green) dual labelling in (**a**) a trabecula and (**b**) ventricle wall sample from failing hearts. (**c**) Co-localisation analysis reveals the extent of RyR co-localised with JPH2 and JPH2 co-localised with RyR in the trabeculae and ventricle wall from failing human hearts. (**d**) Tissue labelling density revealed no differences for RyR or JPH2. (**e**) A positive relationship is observed between JPH2 tissue density and peak stress development (normalised to MCSA) in trabeculae from the failing human heart (p = 0.041). (**f**) JPH2 tissue labelling density forms a strong, positive relationship with the percentage of RyR clusters associated with t-tubules (tt) in failing human trabeculae (p = 0.0014). Co-localisation analysis: Trabeculae: n = 21 images, 14 trabeculae, 5 hearts; failing wall n = 22 images, 5 hearts. Density analysis: Trabeculae: n = 14 images, 14 trabeculae, 5 hearts; failing wall n = 14 images, 5 hearts. Data displayed as mean ± SEM.

Dual labelling of RyR and JPH2 was also performed to determine if junctional organisation was implicated in the observed functional variability in trabeculae. Confocal imaging revealed RyR clusters typically appearing in striations throughout the cardiomyocytes, with JPH2 often seen localised to the surface sarcolemma in failing trabeculae (Fig. 5a). A similar pattern of localisation was observed in failing human ventricular myocardium, with an overall organisation of transversely aligned clusters for both proteins (Fig. 5b). Between the failing samples, there was no difference in the extent of co-localisation observed, with the proportion of JPH2 co-localised with RyR unchanged across the sample groups (Fig. 5c). There was no significant difference in the fraction of RyR co-localised with JPH2 in the failing ventricle myocardium compared to non-failing (Supplementary Fig. S2).

There have been reports suggesting that the expression levels of RyR and JPH2 are decreased in the failing heart^7,23,42,43^, however, our analysis of protein labelling density in the tissue indicates that this was also not altered between our sample groups (Fig. 5d, Supplementary Fig. S2). While the degree of association between these two proteins appeared to vary between trabeculae, neither the extent of RyR co-localised with JPH2 or vice versa was significantly correlated with peak stress production (Supplementary Fig. S3). The density of tissue labelling for both RyR and JPH2 showed positive relationships with peak stress, with JPH2 labelling density revealing a significant correlation (Supplementary Fig. S3, Fig. 5e). Of greater interest was the identification of a strong correlation between increasing tissue JPH2 density and the proportion of RyR clusters associated with the t-tubules (Fig. 5f), indicating a relationship between increased JPH2 levels and an increased number of junctions.

### Microtubule densities in human HF

**Figure 6:**
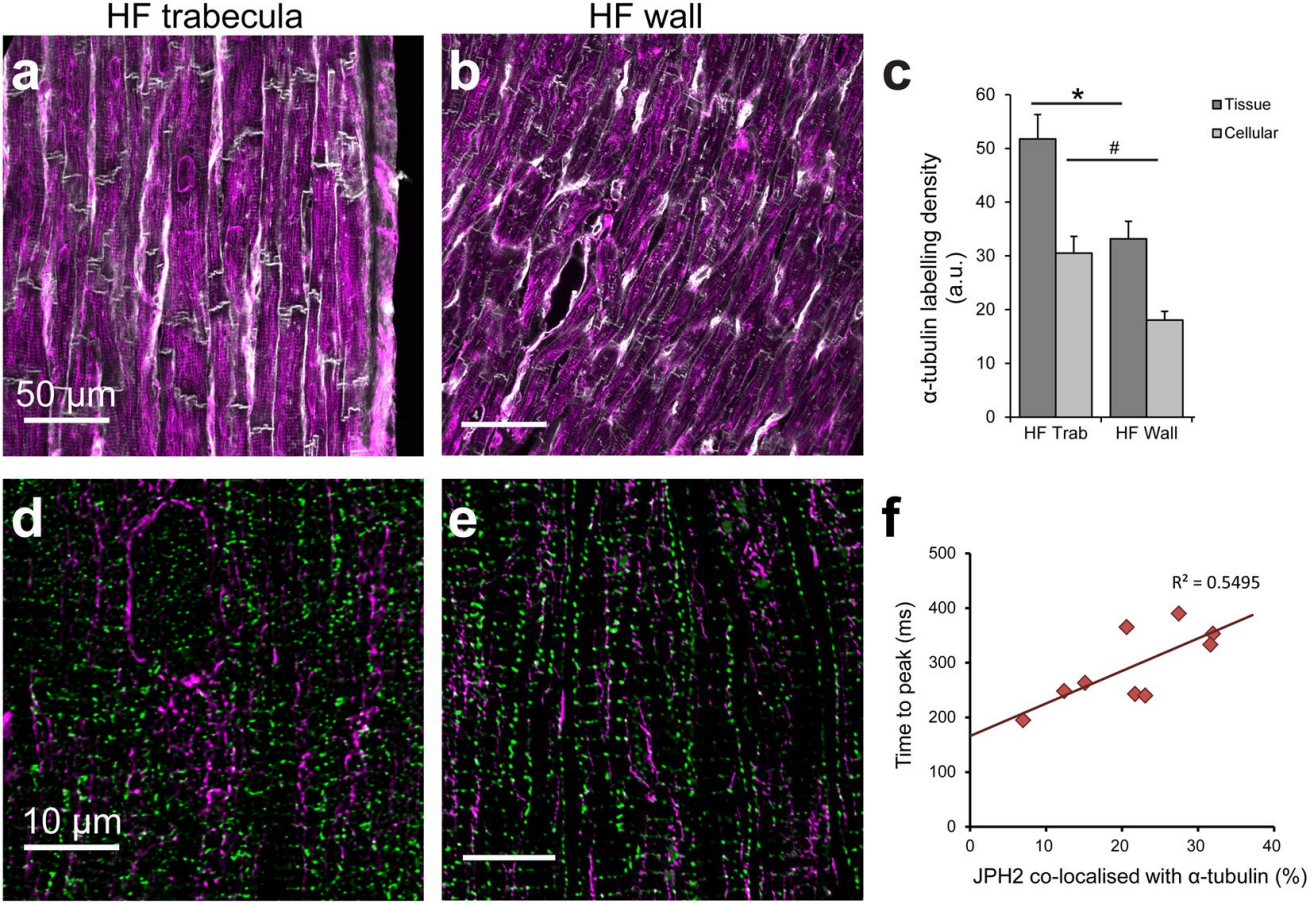
α-tubulin labelling and co-localisation with JPH2. Confocal images of α-tubulin (magenta) in (**a**) failing trabeculae and (**b**) failing ventricle wall, with cell boundaries shown by WGA (grey). (**c**) Density analysis of α-tubulin labelling at both the tissue and cellular level in the failing tissue sample groups. Tissue density analysis: Trabeculae: n = 14 images, 14 trabeculae, 5 hearts; failing wall n = 15 images, 5 hearts. Cellular density analysis: Trabeculae: n = 24 images, 14 trabeculae, 5 hearts; failing wall n = 27 images, 5 hearts. Data displayed as mean ± SEM. *p = 0.03; ^#^p = 0.01. Deconvolved confocal images showing dual labelling with JPH2 (green) in (**d**) a trabecula and (**e**) ventricular myocardium from the failing heart. (**f**) The percentage of JPH2 co-localised with α-tubulin was strongly correlated with increased time-to-peak stress in failing trabeculae (p = 0.017).

Densification of the microtubules has been widely reported in DCM^27–29^ and we therefore investigated this in our samples. Confocal imaging of α-tubulin labelling revealed widespread microtubule presence within and between cardiomyocytes for failing trabeculae (Fig. 6a), as well as the failing ventricular wall (Fig. 6b). Dense networks of microtubules were observed in cardiomyocytes from failing trabeculae and ventricular myocardium (Fig. 6d,e), often including envelopment around intracellular structures presumed to be the nuclei^28^. Analysis of α-tubulin labelling density showed that, when averaged over the whole tissue, there was a significant increase in density in the failing trabeculae compared to the failing ventricular myocardium, as well as when assessed at the cellular level (Fig. 6c). In the non-failing ventricular myocardium, labelling between myocytes was more prominent, with a ~2.8-fold greater density of α-tubulin in the failing ventricular cardiomyocytes compared to non-failing at the cellular level (Supplementary Fig. S2). These density analyses support previous findings of microtubule densification in the failing heart and also reveal the extent of increased α-tubulin within the cardiomyocytes. While there was significant densification of microtubules observed in the failing trabeculae, the density of α-tubulin labelling at neither the tissue nor myocyte level was significantly correlated with peak stress development (Supplementary Fig. S4).

It has been suggested that the densification of microtubules is implicated in increased trafficking of JPH2 towards the surface sarcolemma, away from the junctions, resulting in impaired EC coupling in HF^30^. Dual labelling of JPH2 and α-tubulin revealed varying extents of association between the two proteins, which appeared to be higher in the two failing tissue sample groups (Fig. 6d,e) compared to the non-failing myocardium (Supplementary Fig. S2), but was not statistically significant. While co-localisation analysis of these images revealed significantly increased JPH2-α-tubulin association in the failing trabeculae compared to failing ventricle myocardium, there was no significant relationship identified with peak stress development in the trabeculae (Supplementary Fig. S4). However, a significant correlation was found between the fraction of JPH2 co-localised with α-tubulin and time-to-peak stress in the trabeculae from human HF hearts (Fig. 6f).

### Variable force-frequency relationship in HF trabeculae

Unlike the strong, positive force-frequency relationship (FFR) observed in the healthy human heart, a blunted or even negative FFR has been reported in human HF^33,44^. Examining peak stress in the failing trabeculae across a range of stimulation frequencies (0.1–2 Hz) revealed a wide variability in the FFR from the samples we obtained (see Supplementary Table S1). This included several trabeculae demonstrating the previously reported negative or blunted FFR, while others presented with a clear positive relationship. However, none of the structural parameters examined in this study were identified as significantly correlating with the FFR (data not shown). It should, however, be noted that the FFR was not able to be determined in all trabeculae due to insufficient force development at 1 Hz, preventing identification of a response to altered stimulation frequency.

## Discussion

DCM is among the most common forms of heart disease^45^ and is the most prevalent underlying cause of heart transplant procedures^46^. In this study, we have observed and quantified both tissue level sub-cellular structural changes to IDCM failing human myocardium, as well as explored the relationship between these changes and cardiac function. The defining feature of HF is the impairment of cardiac function, such that there is inadequate supply to meet metabolic demand. While, on average, we observed low active stress development in the trabeculae from failing human hearts, some trabeculae exhibited contractile strength at levels similar to those reported in trabeculae from the healthy human heart^33^. The contractile performance was strongly linked to a number of marked structural alterations at tissue and cellular levels. Importantly, the observed variability in contractile performance and myocyte content did not merely reflect differences between hearts as contractile performance also varied greatly between trabeculae from the same hearts.

Myocardial fibrosis is well established in the diseased and failing heart^2^, with increased collagen deposition leading to higher ventricle wall stiffness and opposition to active force development^3^. Our data appear to support these previous findings, showing a reduction in the proportion of myocardium attributed to cardiomyocytes in the failing human heart (Fig. S4), indicating greater ECM content. Although this did not quite reach statistical significance, this most likely reflects the limited number of non-failing samples in our study. The proportion of tissue attributed to cardiomyocytes was highly varied between trabeculae (Fig. 1), even within a single heart. It was also identified that the variability in myocyte content within failing hearts was greater in the trabeculae compared to ventricular myocardium (Fig. 3). This suggests that cardiac trabeculae may be more susceptible to pathological remodeling of the ECM, although in the absence of non-failing trabeculae samples it is difficult to determine if this is due to natural variation in trabeculae composition, or a result of fibrotic remodeling in HF. Reduced myocyte content was directly implicated in functional impairment in HF trabeculae, with a strong correlation identified between MCSA and active force development (Fig. 1). While increased collagen content of trabeculae has been observed in the spontaneously hypertensive rat model of heart failure, which was associated with reduced active stress^37^, the current study clearly demonstrates myocardium composition to be a strong indicator of peak force development in the failing human heart.

Our study utilised field stimulation to initiate EC coupling, which would make the effects of fibrosis and reduced myocyte content on electrical propagation less obvious as compared to the situation *in vivo*. *In vivo*, the spread of action potentials relies on cell-cell signaling via gap junctions between cardiomyocytes. In that scenario, the increased separation of myocytes with fibrosis and myocyte loss may contribute to a reduced synchronicity between cardiomyocyte activation, thereby leading to a further reduction in peak stress development.

Given that the cardiomyocytes are responsible for the development of active force, it is not entirely surprising to identify this relationship. However, our findings suggest that the classical method of normalising force to total CSA is insufficient to obtain measurements of stress for comparison between failing muscle samples. This classical view was based on findings from healthy muscle, which suggested that CSA was a direct measure of the number of muscle fibers present. Our present study shows that for failing human myocardium, force does not necessarily form a strong relationship with total CSA. We therefore propose that force should routinely be normalised to MCSA to obtain an indication of myocardium contractility, rather than total CSA, particularly in the case of studies utilising failing samples. This would necessitate tissue structure investigation, in addition to force measurements, be regularly performed in such studies.

In the healthy rat heart, a relationship between trabecula CSA and tissue composition has been previously demonstrated, with smaller diameter trabeculae presenting higher collagen content^41^. Such a relationship was not observed in our study of failing human trabeculae (Fig. 1). In the absence of non-failing human trabeculae, it is difficult to distinguish if this is due to normal species differences in tissue composition, or due to pathological changes in these samples resulting in myocyte loss. In non-ischemic heart disease it is unclear as to what triggers ECM proliferation, as it has been reported in the absence of necrotic cell death^47^. Our study found the ECM of trabeculae to be highly positive for collagen – specifically types I, III and VI (Fig. 2). DAPI staining revealed nuclei throughout the extra-cardiomyocyte regions (Fig. 2), indicating the presence of additional cell types distributed throughout the ECM. These likely include activated myofibroblasts which are involved in the deposition of collagen into the ECM^3^ and exhibit positive labelling for α-tubulin^48^, consistent with our observation of α-tubulin in non-myocyte regions (discussed below). An additional observation was the inconsistency of cardiomyocyte orientation in several trabeculae (Fig. 2). The synchronized contraction of myocytes in parallel alignment should allow for maximal force development, therefore myocytes orientated at different angles within a single trabecula would lead to reduced peak force development. This also means that if there is misalignment of myocytes within the trabecula, the actual force able to be developed by these myocytes would be higher than the force measured along a single axis of the trabecula. However, even when factoring in a moderate degree of myocyte misalignment, similar to that observed in our samples, this misalignment would not significantly account for the remaining variability in developed force by the failing trabeculae.

The coupling of the jSR with the t-tubule membrane is required for the formation of junctions^15^, which are essential structures for efficient EC coupling and control of local ion concentrations^49^. We observed a significant reduction in the association between RyR clusters (localised to jSR) and the t-tubules in the failing human heart (Supplementary Fig. S2). Despite maintaining cell-wide organisation, decreased association of RyR clusters with the sarcolemma has also been previously reported in a canine model of HF^50^. This indicates that the reduced RyR cluster association is primarily a result of the well-characterized t-tubule network remodeling in HF^7,12,24^. The displacement of t-tubules from the z-disks leads to increased distances across the junctional cleft and subsequently reduced efficiency of EC coupling and dysynchrony of the Ca^2+^ transient^11^, typically resulting in impaired cardiac function. However, we found that, while a positive trend was present, RyR-t-tubule association was not significantly correlated with active stress when normalised to cardiomyocyte content in failing human heart trabeculae.

Interestingly, we did identify a significant correlation between the myocyte content of trabeculae and the proportion of RyR clusters associated with t-tubules (Fig. 4). This suggests the possibility that increased myocyte content of the tissue could be protective of intracellular remodeling in HF. Increased fibrosis leads to increased stiffness of the ventricle wall and therefore higher passive tension in the tissue. The resistance against which the myocytes are required to contract in order to develop active force increases^2^ and subsequently, a greater active force (stress) is necessary for myocyte shortening to occur. In an elastic material, the strain developed is proportional to the applied stress and it has been shown that exposure to increased strain leads to remodeling of t-tubules in cardiomyocytes^51,52^. This provides a potential mechanism through which increased fibrosis and a corresponding decrease in cellular content, may contribute to the reduced association between RyR clusters and t-tubules in the remaining cardiomyocytes.

The association between JPH2 and RyR has been suggested to modulate the open probability of the Ca^2+^ channel and impact Ca^2+^ spark properties^18,20^, contributing to spontaneous RyR opening and raised diastolic Ca^2+^, as observed in HF. However, we found no significant difference in either the RyR-JPH2 association in the failing heart compared to non-failing, nor in the labelling density of these proteins, despite previous studies reporting reduced protein expression levels reported^23,26,42,43^. However, JPH2 tissue density was positively correlated with increased association between RyR clusters and t-tubules in the trabeculae (Fig. 5) – further supporting the importance of JPH2 in the formation and maintenance of cardiac junctions.

As commonly reported in HF, densification of microtubules was observed in the failing myocardium, both within and between cardiomyocytes (Fig. 6). Microtubule staining observed between myocytes is likely to indicate the presence of smooth muscle cells of the capillaries^53^, or activated myofibroblasts^48^ (which are involved in deposition of collagen in the ECM^3^). It has been proposed that microtubules potentially modulate Ca^2+^ currents through both DHPR and RyR^54,55^, as well as their densification increasing resistive stresses within the myocyte, inhibiting sarcomere shortening^56^. Despite these proposed mechanisms, α-tubulin density was not correlated with active force development in the human HF trabeculae (Supplementary Fig. S4).

Another suggested role of microtubules in HF is the altered trafficking of JPH2, whereby redistribution of JPH2 away from junctions may impair the ability to maintain junctional organisation and potentially lead to reduced EC coupling gain across the junctional cleft^30^. While increased co-localisation between α-tubulin and JPH2 was identified in the failing human trabeculae, this was not found to be correlated with the peak active stress (Supplementary Fig. S4). It is possible that it is not the direct interaction of JPH2 with microtubules that leads to functional impairment, but instead the end distribution of JPH2 following peripheral trafficking. That being said, the fraction of JPH2 co-localised with α-tubulin was found to be positively correlated with time-to-peak stress, such that a longer time was required to reach peak active stress. As time-to-peak stress in this study was measured from stimulus onset, this parameter incorporates the time required for EC coupling to occur, as well as cross-bridge cycling rates. Increased association of JPH2 with microtubules may reduce its ability to maintain both junctional and t-tubule organisation. Consequently, desynchronisation of the action potential can occur as a result of t-tubule remodeling, leading to impairment of EC coupling and potentially the increased time-to-peak stress.

It is currently an open question if the great variability in myocyte content is a result of cardiac remodeling in failure (as we would speculate) or perhaps a more general property of human cardiac trabeculae also found in normal hearts. While the lack of trabeculae from non-failing human hearts is a limitation, it is notable that our comparison between trabeculae from failing hearts allowed circumvention of potentially confounding pharmacological differences between patient treatment regimes by using multiple trabeculae from each heart to provide within-heart comparisons.

A commonly reported alteration in HF are changes to the Ca^2+^ transient, including the amplitude and temporal kinetics, which are thought to be involved in alterations of the FFR. As a result of limited trabeculae numbers and the time required for the force experimental protocol, the number of trabeculae able to be loaded with Ca^2+^ indicator in this study (n = 3) was insufficient for a systematic investigation of changes to Ca^2+^ handling in human HF; accordingly, we cannot currently dissect the reason for the variability of FFR in our samples. The blunted, or even negative, FFR in the failing heart is also thought to involve a shift towards Ca^2+^ extrusion via NCX, such that there is an increasing removal of Ca^2+^ from the myocyte with higher stimulation frequencies^57,58^. It would therefore be of interest to examine both the function and distribution of NCX and SERCA in relation to the FFR in further studies. While direct correlation between force, structure and Ca^2+^ handling in individual trabeculae from human hearts would be of great interest to pursue in future studies – it is clearly technically very challenging.

In conclusion, using a novel approach, we obtained both functional and structural measurements in individual muscle samples from the failing human heart. This enabled us to determine structural parameters directly correlated with functional variability, both within and between hearts. We observed highly variable cardiomyocyte content in trabeculae from failing human hearts, which was strongly correlated with contractile function, while total tissue CSA was not, contrary to assumptions generally made in trabeculae studies. Trabeculae from healthy hearts are almost entirely made up of myocytes^59^, such that force normalised to total CSA still remains acceptable for comparison between muscles. However, measurements from hypertrophied or failing hearts may over-estimate the degree of dysfunction unless force is normalised to MCSA. On this basis, we suggest that force studies should be augmented with structural assessments in order to draw mechanistic conclusions, particularly in HF studies. In addition to these tissue-level changes, several sub-cellular protein changes were identified and confirmed as occurring in HF compared to non-failing hearts. Trabeculae structural properties broadly mimicked features of the ventricular wall from the same hearts, confirming their usefulness in functional studies, particularly if effects of structural variability are eliminated by correlation with functional measurements.

## Materials and Methods

### Ethics and sampling

Ethical approval was obtained from the New Zealand Health and Disabilities Ethics Committee (NTY/05/08/050) for the collection of human samples, along with written, informed consent of the heart transplant recipients and the families of organ donors in the case of non-failing tissue samples. No samples were procured from prisoners, with all experiments performed in accordance with relevant guidelines and regulations. Samples from the septal and ventricular walls were collected from the explanted hearts of patients with non-ischemic IDCM in end-stage HF from Auckland City Hospital. These were immediately transported on ice in oxygenated Tyrode’s solution containing 0.25 mM CaCl_2_+20 mM 2,3-butanedione monoxime (BDM; Sigma-Aldrich) to the laboratory. Suitable cardiac trabeculae were then micro-dissected from the endocardial surface of tissue blocks for functional experiments (see Supplementary Fig. S1). Free-wall tissue from regions adjacent to trabeculae sampling was retained for tissue structure analysis. During this study, non-failing hearts explanted at Auckland Hospital were all utilised for heart transplantation and therefore collection of non-failing cardiac trabeculae was not possible; however, previously obtained free wall tissue samples, stored at −80 °C, from non-failing control hearts of unmatched organ donors were utilised in some immunolabelling experiments. Echocardiogram data obtained before organ explantation confirmed that non-failing hearts presented with ejection fractions (EF) within the accepted normal range^60^, while failing hearts were significantly impaired, with EF values ranging from 9–27%. Patient details are summarised in supplementary Table S2.

### Functional experiments and data analysis

Suitable trabeculae were identified according to length and diameter (mean diameter ± SEM: 330 ± 150 µm) such that adequate oxygen diffusion could be maintained, based on tissue eccentricity^61^ and were comparable to sizes used in previous studies^31,34,36^. The experimental setup and procedures were adapted from those previously described^37,62^. In brief, dissected trabeculae with small ventricular tissue blocks on each end were transferred to a tissue bath positioned on the stage of an inverted Nikon Eclipse TE2000 microscope. Trabeculae were held at fixed length (L_O_) by mounting the tissue block at one end in a wire cradle which extended from a force transducer (KX801 Micro Force Sensor, Kronex Technologies), while the tissue block at the opposite end was secured in a nylon loop. Both ends were attached to manipulators to enable control of muscle length. Trabeculae were continuously superfused with oxygenated Tyrode’s solution containing 2 mM [Ca^2+^]_o_, and 0 mM BDM at 37 °C, unless otherwise stated. Samples were field stimulated with supramaximal 5 ms electrical pulses at 1 Hz (model D100, Digitimer™, UK). Data was acquired using LabVIEW (National Instruments, Austin, USA) and analysed offline.

The peak twitch force was measured as the difference between the diastolic force and the peak force developed at 1 Hz stimulation. This was averaged across five sequential steady state contractions from each trabecula using Excel (Microsoft Corp.). A total of 14 trabeculae were analysed from the five failing hearts. It has been long recognized that developed force is proportional to muscle size, in particular cross-sectional area (CSA)^40^. Force measurements were therefore normalised to trabecula CSA and expressed as stress (mN/mm^2^) to enable comparison between trabeculae. Total trabecula CSA was determined by direct measurement from confocal images of fixed tissue transverse sections, rather than calculated from live preparations in which orientation may influence diameter measurements – particularly for elliptical tissue samples. While shrinkage may have occurred through tissue processing, this would be consistent across all samples. Confocal imaging revealed a large variability in the proportion of myocytes between trabeculae, which was found to positively correlate with developed force. Therefore, force was also normalised to myocyte CSA (MCSA) of the trabecula, with both stress per CSA and stress per MCSA determined for all trabeculae. Analyses performed on time-to-peak stress and and time to 50% relaxation (T_50%_), are presented in the supplementary material (see Supplementary Fig. S1).

### Tissue processing and fluorescent labelling

Following dissection of trabeculae, blocks from the remaining tissue were fixed in 2% (w/v) paraformaldehyde for 1 h at 4 °C. Upon completion of functional experiments, the trabeculae were held at fixed length and fixed with the same protocol. After fixation, the trabeculae were cut in half transversely. All samples were cryoprotected in sucrose, frozen in methyl butane cooled in liquid nitrogen and stored at −80 °C, with the trabeculae halves embedded in TissueTek® O.C.T. compound. Frozen 16 µm thick sections of myocardium (failing and non-failing) and trabeculae were cut on a CM 3050 cryostat (Leica, Germany) and collected onto 0.05% poly-L-lysine (v/v) coated coverslips for immunohistochemistry. Immunolabelling procedures were adapted from those previously described^63^. Tissue sections were hydrated in PBS and permeabilised with 1% Triton-X100 (v/v; Sigma-Aldrich) for 10 min at room temperature, followed by blocking in Image-iT Signal enhancer (Life Technologies) for 1 h at room temperature. Immunolabelling was performed using rabbit anti-JPH2 (1:100, custom polyclonal, as previously detailed)^20,64^ combined with either mouse anti-RyR (1:100, ma3-916, Thermo) or mouse anti-α-tubulin (1:200, Ab184613, Abcam) as a microtubule marker^53^, or rabbit anti-collagen I (1:200, ab292, Abcam) or anti-collagen VI (1:100, ab6588, Abcam) with mouse anti-collagen III (1:200, ab6310, Abcam) with the primary antibodies incubated overnight at 4 °C. Sections were washed and incubated with goat anti-mouse and goat anti-rabbit secondary antibodies conjugated to Alexa Fluor 488 or 568 (1:200, Life Technologies). T-tubules and ECM were labelled using wheat germ agglutinin^63,65,66^ Alexa Fluor 647 (WGA; 1:200, Life Technologies), applied concomitantly to the secondary antibodies for 2 hours at room temperature. Collagen-labelled sections were also incubated with either DAPI (1:50, Life Technologies) to stain nuclei, or phalloidin Alexa Fluor 647 (1:50, Life Technologies) to stain f-actin, concomitantly with secondary antibodies. All antibodies were dissolved in 1% BSA (w/v) + 0.05% NaN_3_ (w/v) + 0.05% Triton-X100 (v/v) in PBS. Samples were mounted onto slides using ProLong Gold (Life Technologies) and allowed to cure for ~72 h prior to imaging.

### Confocal microscopy and image processing

A Zeiss LSM710 inverted confocal microscope was used to acquire images of the fluorescently labelled tissue sections using either a 40 × NA 1.3 oil-immersion or a 63 × NA 1.4 oil immersion objective. 8-bit images were generated with 173 µm/pixel or 67 µm/pixel, respectively. Fluorochromes were excited with either 405 nm (DAPI), 488 nm (Alexa 488), 561 nm (Alexa 568) or 633 nm (Alexa 647) laser excitation. Images were acquired of both longitudinally and transversely orientated cardiomyocytes, with confocal image stacks processed for deconvolution according to previously described protocols using a Richardson-Lucy algorithm^14^ to produce 32-bit image stacks. Tissue content was assessed in images of transversely orientated cardiomyocytes (acquired at 40× magnification) in all three sample groups, using ImageJ. Manual thresholding was applied to generate binary masks. JPH2 labelling was used to determine MCSA, while WGA labelling was used to measure total CSA. The outline of each WGA mask was manually traced to determine the total CSA of the trabecula tissue sections. Within each individual trabecula transverse section, all regions positive for JPH2 staining were combined to determine the CSA attributed to myocytes (MCSA). MCSA was also converted to a percentage of total CSA.

The extent of RyR-t-tubule association was assessed in RyR and WGA dual-stained images (acquired at with the 63× objective). Binary masks of labelling-positive regions were generated following thresholding using the value calculated from mode plus standard deviation of the grayscale image pixel values in ImageJ. This thresholding method used the algorithm described by Sachse *et al*.^50^. Briefly, the threshold was calculated from image statistics, i.e. the intensity histogram and and set to its mode+ one standard deviation. ImageJ particle analysis was used to identify RyR clusters as regions of interest (ROI), which were then overlaid on the corresponding WGA mask. The number of RyR clusters associated with t-tubule labelling could then be determined as ROI with mean pixel value > 0 (i.e. ROIs with overlap with the t-tubule mask) and we report the fraction of t-tubule positive ROIs as the percentage of total RyR clusters present.

Co-localisation analysis was performed on the images of longitudinal cardiomyocytes that were dual labelled for JPH2-RyR and JPH2-α-tubulin, using previously established methods with custom written scripts in Python software^67^. In brief, RyR-JPH2 dual-labelled images were thresholded using the mode plus standard deviation value in ImageJ, while JPH2-α-tubulin images had isodata thresholding applied and masks were generated of each protein label with Python software. From each protein label, the distance to the nearest labelling of the second protein mask was determined using distance maps to generate a distance distribution plot. Distances up to and including zero were summed as a measure of the fraction of protein co-localisation. Since WGA staining was used to identify both the t-tubules and ECM, this co-localisation method would not have been suitable for determining the association between RyR and t-tubules, as it would indicate the fraction of total WGA labelling associated with RyR, rather than t-tubules specifically.

Labelling density of α-tubulin was assessed to determine the degree of microtubule densification. This was achieved by applying isodata thresholding to longitudinally orientated cardiomyocyte images (acquired with 40x magnification) to generate a binary mask. The fractional area of the total tissue section covered by the mask was determined and used as normalised global tissue labelling density measurement. As vascular smooth muscle cells have also been shown to express α-tubulin^53^, the corresponding analysis was performed following selection of intra-cardiomyocyte regions (in 63x magnification-acquired images) to obtain cellular labelling density. Tissue labelling density was also assessed for RyR and JPH2.

### Statistical analyses

Statistical tests were performed using IBM SPSS Statistics v22 or SAS statistical package, with p < 0.05 considered significant. Pearson’s correlation analysis was performed on variables for cardiac trabeculae functional experiments. Differences in structural parameters between failing trabeculae and ventricle wall samples were analysed as a triply-nested design using the Generalised Linear Model (GLM) of the SAS statistical package. Variability within the data arising from each dependent variable of interest (Relative Myocyte Area, for example) was partitioned between ‘Group’ (‘trabeculae’ versus ‘ventricles’) and ‘Region’ (‘septum’, ‘LV’ and ‘RV’) and accounted for multiple samples from the same heart and same region using a third independent variable labelled ‘repetition’. Explicitly, ‘Hearts’ (n = 5) were nested with ‘Group’, ‘Region’ was nested within both ‘Group’ and ‘Heart’ and ‘repetition’ was nested within ‘Group’, ‘Heart’ and ‘Region’. This design accounted for all of the variability in the data, returning a value of zero for Error and a value of unity for r^2^. Since the design was unbalanced, statistical significance was examined (P < 0.05) using the Type III Sum of Squares. Differences in myocyte content were also evaluated with the same design except ‘Group’ had three categories (‘trabeculae weak’, ‘trabeculae strong’ and ‘ventricular’). In this design p-values are reported for post-hoc procedure using a mutually-orthogonal set of contrast coefficients. Means are reported with standard error of the mean (SEM) as uncertainty, unless otherwise stated. All n-numbers are reported in corresponding figure legends.

### Data availability

All datasets generated during and/or analysed during the current study are available from the corresponding author on reasonable request.

## Electronic supplementary material

Supplementary Information
