# Supplementary material for: Highly variable contractile performance correlates with myocyte content in trabeculae from failing human hearts
Source: Sci Rep. 2018 Feb 13;8:2957. doi: 10.1038/s41598-018-21199-y (PMC5811450; doi:10.1038/s41598-018-21199-y)
Supplement: Supplementary file 1 — Supplementary Information [file 41598_2018_21199_MOESM1_ESM.pdf]

Highly variable contractile performance correlates with myocyte content in trabeculae from failing human hearts

Michelle L. Munro, Xin Shen, Marie Ward, Peter N. Ruygrok, David J. Crossman & Christian Soeller

Supplementary Material

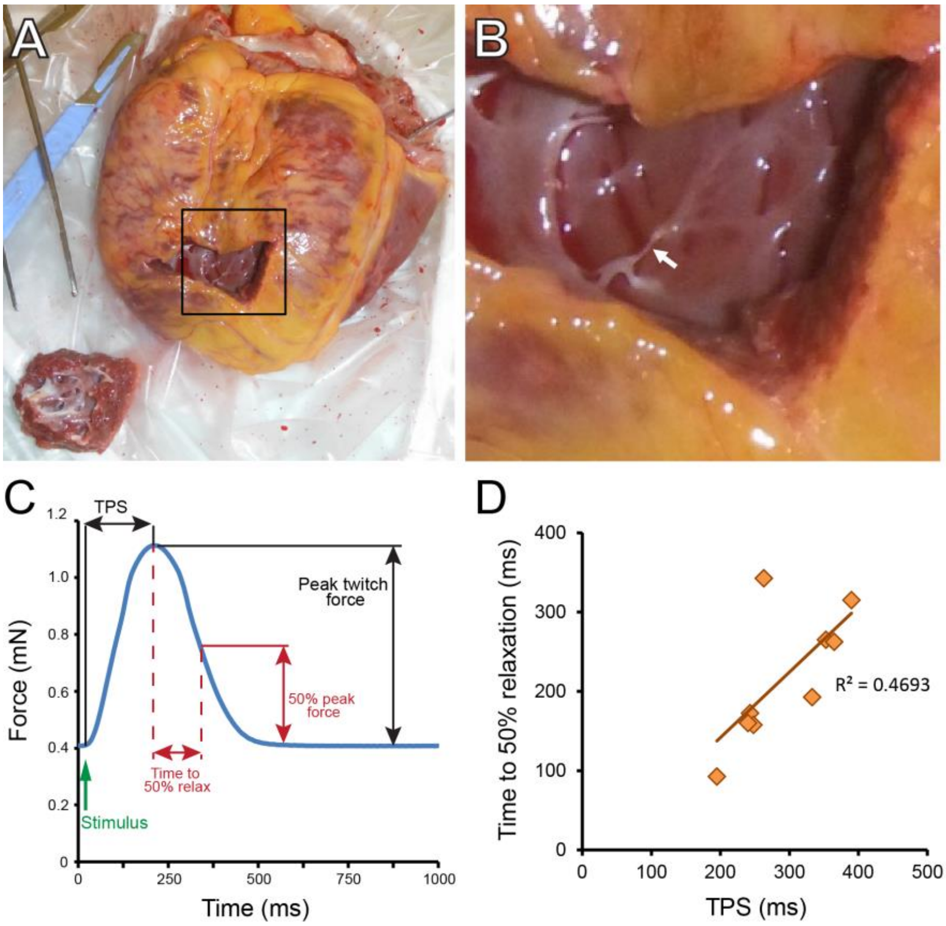

Fig S1. Trabeculae dissection and functional analyses

A) Explanted human heart with IDCM after the removal of left ventricular wall sample shown, with B) an underlying trabecula on the septal wall prior to dissection (indicated with arrows). C) Method of functional data analysis, indicating how time to peak stress (TPS), peak twitch/active force and time to 50% relaxation ($T_{50\%}$) were measured. D) Correlation analysis revealed a positive relationship between TPS and $T_{50\%}$.

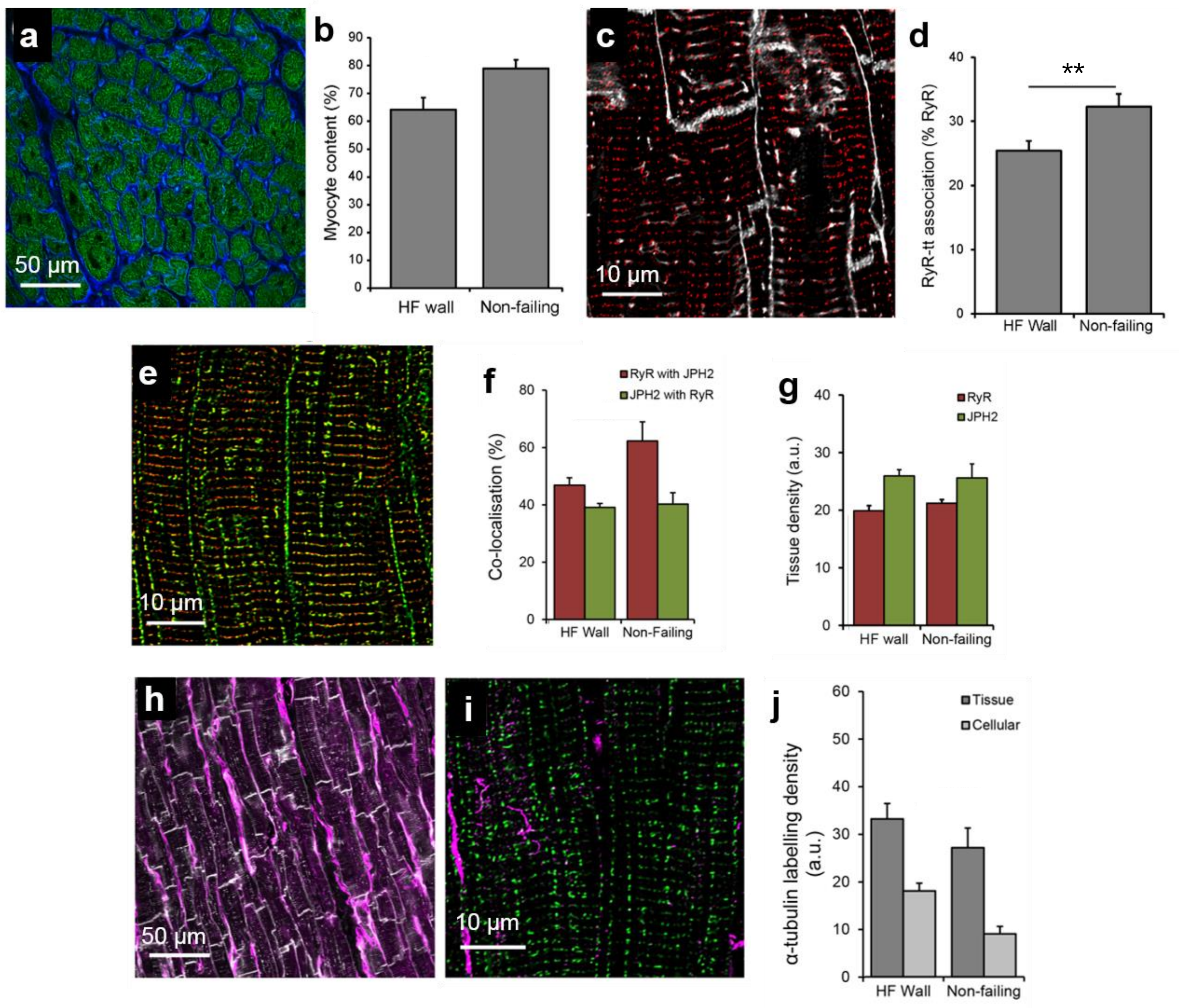

Fig S2. Structural analyses in non-failing human ventricular myocardium

A) Confocal image showing the tissue composition in ventricular myocardium from non-failing heart with ECM (WGA; blue) and JPH2 (green) labelling. B) Analysis of tissue composition in the failing and non-failing ventricle wall C) Deconvolved confocal image showing t-tubule (WGA; grey) and RyR (red) dual labelling in cardiomyocytes from non-failing ventricle. D) Analysis of RyR-t-tubule association was performed in the failing and non-failing human myocardium. E) Deconvolved confocal micrographs showing RyR (red) and JPH2 (green) dual labelling in ventricle wall sample from non-failing heart. F) Co-localisation analysis of RyR and JPH2 in the ventricle wall from failing and non-failing human hearts. G) Tissue labelling density for RyR and JPH2. H) Confocal image of α-tubulin (magenta) in non- failing ventricle wall, with cell boundaries shown by WGA (grey). I) Deconvolved confocal image showing dual labelling with JPH2 (green) in non-failing ventricular myocardium. J) Density analysis of α-tubulin labelling at both the tissue and cellular level. Myocyte content analysis: non-failing wall n = 6 images, 3 hearts. Co-localisation analysis: non-failing wall n = 6 images, 3 hearts. JPH2/RyR Density analysis: non-failing wall n = 6 images, 3 hearts. Tubulin tissue density analysis: non-failing wall n = 6 images, 3 hearts; cellular density analysis: non-failing wall n = 7 images, 3 hearts. Data displayed as mean ± SEM. **p=0.0044.

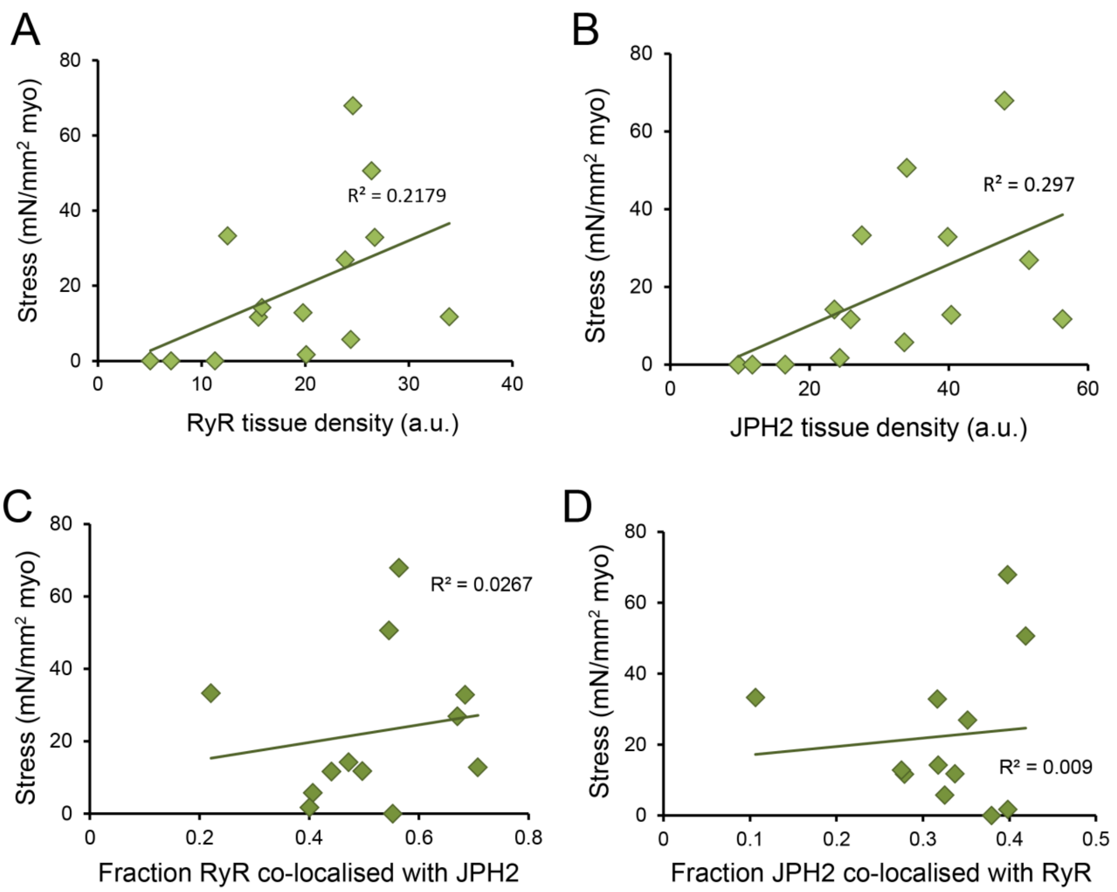

Fig S3. Correlation analyses of stress with RyR and JPH2 density and co-localisation

A) RyR tissue labelling density was not significantly correlated with stress (normalised to MCSA), while B) JPH2 tissue labelling density was. Co-localisation analysis revealed neither C) the fraction of RyR co-localised with JPH2 or D) fraction of JPH2 co-localised with RyR were correlated with peak stress production in trabeculae from the failing human heart.

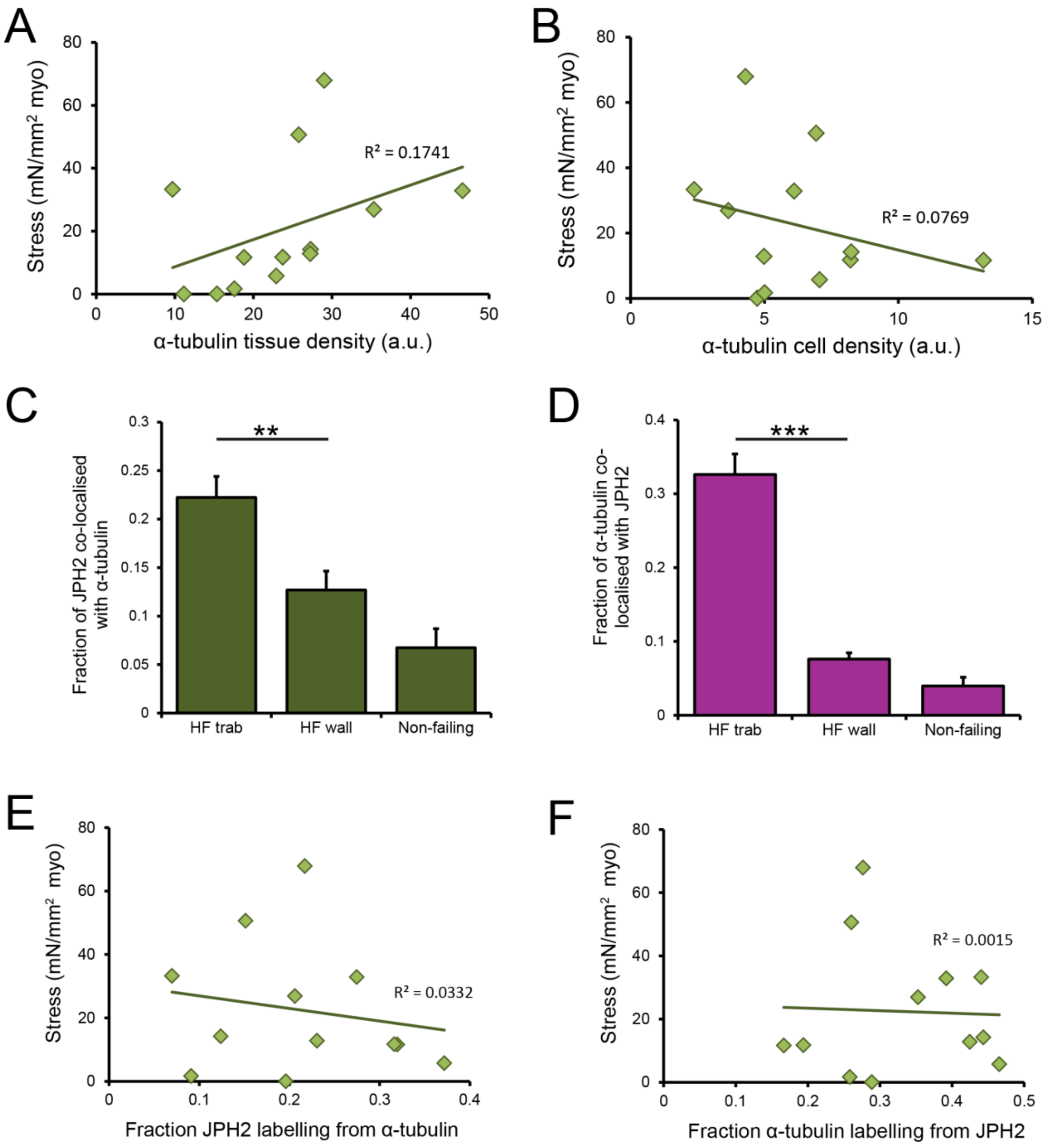

Fig S4. Analyses of α-tubulin density and JPH2 co-localisation correlation with stress

A significant correlation was not observed between active stress development (normalised to MCSA) and α-tubulin labelling density at either A) the tissue or B) cellular level. Co-localisation analysis revealed both C) the fraction of JPH2 co-localised with α-tubulin and D) the fraction of α-tubulin co-localised with JPH2 were increased in the failing trabeculae and ventricular wall samples. Neither E) the fraction of JPH2 co-localised with α-tubulin nor J) the fraction of α-tubulin co-localised with JPH2 were correlate with peak stress. Co-localisation analysis: Trabeculae: n = 23 images, 14 trabeculae, 5 hearts; failing wall n = 14 images, 5 hearts; non-failing n = 6 images, 3 hearts. Data displayed as mean ± SEM; $^{**}p<0.01$, $^{***}p<0.001$.

Heart	Trab	Force (mN)	CSA (mm^2)	MCSA (mm^2)	Stress (mN/mm^2)	TPS (ms)	$T_{50\%}$ (ms)	FFR gradient ($mN/mm^2/Hz$)
**DH32**	RV3*	N/A	0.128	0.083	N/A*	N/A*	N/A*	N/A
**DH32**	Sept3	1.29	0.209	0.111	7.76	353	270	-3.33
**DH36**	RV3	0.15	0.073	0.026	2.05	N/A	N/A	N/A
**DH36**	RV4	3.26	0.185	0.121	30.30	365	263	-2.25
**DH37**	LV3	0	0.137	0.001	0	N/A	N/A	N/A
**DH37**	RV2	0.32	0.037	0.028	9.38	333	193	-6.05
**DH37**	Sept2	0	0.044	0.003	0	N/A	N/A	N/A
**DH37**	Sept3	0	0.077	0.001	0	N/A	N/A	N/A
**DH38**	RV1	1.35	0.032	0.20	42.99	243	173	+26.47
**DH38**	RV2	0.10	0.021	0.007	11.12	248	156	+3.97
**DH38**	Sept1	0.16	0.011	0.005	13.93	195	93	+1.85
**DH38**	Sept2	0.95	0.131	0.074	8.54	240	160	+4.06
**DH40**	RV1	1.90	0.083	0.058	22.83	390	315	+3.56
**DH40**	RV2	0.01	0.052	0.006	0.189	N/A	N/A	N/A
**DH40**	Sept1	2.20	0.065	0.043	29.51	263	343	+4.15
**Mean**		0.835±0.28	0.086±0.015	0.039±0.011	12.76±3.65	292±23	218±28	+3.60±3.12

Table S1. Variability of contractile performance in failing cardiac trabeculae

Summary of contractile performance parameters measured in 15 trabeculae from failing human hearts, including patient heart ID number, trabecula number from heart (RV = right ventricle, LV = left ventricle, sept = septum). Parameters measured include peak twitch force production at 1 Hz stimulation (at 37°C, 1.5 mM $[Ca^{2+}]_o$), cross-sectional area (CSA) of trabecula, myocyte cross-sectional area (MCSA), normalised stress development, time to peak stress (TPS) and time to 50% relaxation ($T_{50\%}$). The gradient from FFR analysis is also included based on peak stress measurements. Bottom row shows averaged data from trabeculae displayed as mean ± SEM. N/A = peak twitch force was too low to accurately measure parameter. *Trabecula exhibited ectopic contractions so data was not collected at 1 Hz.

Heart ID	Age	Gender	Diagnosis	EF (%)	LVEDD (cm)	LVESD (cm)
**DH32**	58	M	IDCM, NYHA class III-IV	9	8.2	N/A
**DH36**	56	M	IDCM, NYHA class III	25	7.5	6.6
**DH37**	53	M	IDCM, NYHA class III	27	5.2	4.3
**DH38**	23	M	IDCM, NYHA class III	19	8	N/A
**DH40**	66	M	IDCM, NYHA class III	21	7.6	6.9
**N1**	54	F	Normal	74	5.1	2.9
**N2**	62	F	Normal	N/A	4.1	2.3
**N3**	57	F	Normal	N/A	N/A	N/A

Table S2. Patient detail summary

Summary table of patient details, including identification number of the heart, patient age (in years), gender (F = female, M = male), pathological diagnosis (IDCM = idiopathic dilated cardiomyopathy) including New York Heart Association (NYHA) functional classification of failure. Functional indicator parameters include ejection fraction (EF), left ventricular end-diastolic diameter (LVEDD) and left ventricular end-systolic diameter (LVESD). N/A = data not available, but echocardiogram was reported as normal by attending cardiologist for non- HF patients.
